# Artificial-intelligence–based developmental screening for pre-school children (ages 3–6): a scoping review of deployed tools, clinical validity, equity, and implementation in guidance-center settings

**DOI:** 10.3389/fpsyg.2026.1913332

**Published:** 2026-08-20

**Authors:** Adem Arslan, Fatih Gül

**Affiliations:** 1 Department of Child Care and Youth Services, Vocational School of Health Services, Gümüşhane University, Gümüşhane, Türkiye; 2 Artificial Intelligence and Internet of Things Research Laboratory, Department of Electrical-Electronics Engineering, Recep Tayyip Erdoğan University, Rize, Türkiye

**Keywords:** algorithmic equity, artificial intelligence, autism spectrum disorder, developmental screening, digital phenotyping, implementation science, machine learning, pre-school children

## Abstract

**Background:**

Early identification of neurodevelopmental conditions during the pre-school years (ages 3–6) is critical for timely intervention, yet traditional questionnaire-based screeners suffer from low positive predictive value, late detection, and inequitable access. Artificial intelligence (AI) and machine-learning (ML) methods promise more objective, scalable identification, and two devices (Cognoa Canvas Dx, 2021; EarliPoint Evaluation, 2023) are already US Food and Drug Administration (FDA)-authorized. Recent reviews summarize the technical literature, but few address how deployed tools perform on clinical validity, equity, and real-world implementation, particularly outside high-income, English-speaking settings.

**Methods:**

Following Joanna Briggs Institute methodology and the PRISMA extension for scoping reviews (PRISMA-ScR), we searched seven databases (1 January 2020–15 June 2026) and charted primary validation studies of AI/ML developmental-screening tools for children aged 72 months or younger. We mapped tools by modality, diagnostic performance, regulatory status, geographic origin, and, as a structured analytic axis, demographic subgroup (equity) reporting and deployment readiness.

**Results:**

Sixteen primary AI-tool studies met inclusion, spanning eight modality classes (eye-tracking, multimodal questionnaire-plus-video, computer-vision home/clinical video, tablet/smartphone digital phenotyping, voice/acoustic ML, electronic-health-record risk prediction, questionnaire-based ML, and wearable sensors) and eight countries (USA, South Korea, Australia, Switzerland, France, Israel, China, Vietnam). Reported sensitivities ranged from approximately 75 to 99.2% and specificities from 78.9 to 95.5%. Only one tool demonstrated stable performance across sex, race, and ethnicity; subgroup-stratified validation was otherwise near-absent. No CE-marked autism-specific screening device was identified.

**Conclusions:**

The evidence base has broadened in modality and geography but remains autism-concentrated and equity-blind, with a wide gap between analytic accuracy and primary-care deployment. We propose equity validation, deployment-readiness reporting, and an implementation pathway for low-resource guidance-center settings as priorities.

## 1 Introduction

Neurodevelopmental conditions, including autism spectrum disorder (ASD), language delay, attention-deficit/hyperactivity disorder (ADHD) precursors, and global developmental delay, affect a substantial fraction of pre-school-aged children. The United States Centers for Disease Control and Prevention's Autism and Developmental Disabilities Monitoring (ADDM) Network reported an ASD prevalence of 1 in 36 children aged 8 years for surveillance year 2020 (Maenner et al., 2023). Earlier identification translates into earlier intervention and improved cognitive, language, and adaptive outcomes (Zwaigenbaum et al., 2015), and the American Academy of Pediatrics recommends universal ASD screening at the 18- and 24-month visits with ongoing surveillance through the pre-school years (Hyman et al., 2020).

For more than two decades, the dominant primary-care screeners have been parent-completed questionnaires, of which the Modified Checklist for Autism in Toddlers–Revised with Follow-up (M-CHAT-R/F) is most widely deployed. In its pivotal validation in over 16,000 toddlers it achieved sensitivity 0.85 and specificity 0.99, but positive predictive value (PPV) was only 0.48 (Robins et al., 2014): roughly half of positive screens are false positives in low-prevalence settings. The screening-to-diagnosis pipeline also breaks down at referral, with average diagnosis ages exceeding 4 years in many regions and well-documented disparities by race, ethnicity, sex, and socioeconomic status (Pham et al., 2022; Aylward et al., 2021; Cruz et al., 2024).

Against this backdrop, AI and ML approaches, encompassing computer vision, deep learning, eye-tracking analytics, voice processing, tablet/smartphone digital phenotyping, wearable sensing, and electronic-health-record (EHR) risk prediction, have emerged as complementary screening modalities. Two devices hold FDA authorization: Cognoa Canvas Dx (De Novo, June 2021) (Megerian et al., 2022) and EarliPoint (510(k), June 2023) (Jones et al., 2023). Numerous research-stage tools report strong diagnostic-accuracy metrics across toddlers and pre-schoolers (Perochon et al., 2023; Ko et al., 2023; Barbaro and Yaari, 2020).

### 1.1 Why another review, and what this one adds

The AI-autism literature is now actively reviewed. Recent syntheses aggregate dozens to hundreds of research studies and report pooled accuracy ranges: modality-bounded scoping reviews of face/voice/text (Mohammadi et al., 2025) and of voice/behavioral data (Rakotomanana and Rouhafzay, 2025); a systematic review of biomarkers and explainable AI (Agrawal and Agrawal, 2025); a generative-AI scoping review (Sohn et al., 2025); broad narrative sweeps (Shen and Yu, 2025; Zhang, 2025); a fetal-to-adolescent pediatric-AI systematic review (Wang et al., 2025); a child-development monitoring review with an acceptance lens (Reinhart et al., 2024); and a focused review of smartphone apps for ASD screening (Reddy et al., 2024). A narrative review in this field already covers eye-tracking, voice, and video tools and discusses scalability, regulation, and ethics (Patel and others, 2025), and an unreviewed pre-print specifically targets the pre-school band (Hasan, 2025).

We therefore do not claim primacy on the pre-school focus or on cataloging AI methods. Instead, this review is distinguished by three axes that prior syntheses leave largely untouched (Table 1). First, we profile deployed and near-deployed tools as case studies and assess their deployment readiness rather than aggregating method papers on accuracy alone. Second, we treat equity, demographic subgroup-stratified validation, as a first-class, tabulated analytic axis. Third, we situate adoption in a low-resource guidance-center health system, using Türkiye's Guidance and Research Center (Rehberlik Araştırma Merkezi, RAM) network as a concrete implementation exemplar, a context absent from every prior review we identified.

**Table 1:** Positioning against recent reviews of AI for autism / developmental screening (✓, addressed substantively; P, partial/in passing; —, not addressed).

Review	Type	Focus	#st.	Adopt.	Equity	Regul.	LMIC
Patel 2025 (Patel and others, 2025)	Narrative	Eye-track/voice/video, ASD/NDD	—	P	P	P	—
Hasan 2025 (Hasan, 2025)^†^	Systematic	Preschool ASD, AI	—	—	—	—	—
Zhang 2025 (Zhang, 2025)	Narrative	Screening+dx+intervention	—	P	—	—	—
Mohammadi 2025 (Mohammadi et al., 2025)	Scoping	Face/voice/text	23	—	P	—	—
Rakotomanana 2025 (Rakotomanana and Rouhafzay, 2025)	Scoping	Voice/behavioral	158	—	P	—	—
Sohn 2025 (Sohn et al., 2025)	Scoping	Generative AI / LLMs	10	—	—	—	—
Agrawal 2025 (Agrawal and Agrawal, 2025)	Systematic	Biomarkers / XAI	106	—	—	—	—
Shen 2025 (Shen and Yu, 2025)	Narrative	Broad technology sweep	—	P	P	—	—
Wang 2025 (Wang et al., 2025)	Systematic	Pediatric AI, fetal–18	133	P	—	—	—
Reinhart 2024 (Reinhart et al., 2024)	Systematic	Child-dev monitoring, acceptance	—	✓	—	—	—
**This review**	**Scoping**	**deployed tools, eight modalities**	**16**	✓	✓	✓	✓

No prior synthesis combines deployed-tool case studies, a structured equity matrix, a deployment-readiness lens, and a low-resource guidance-center implementation pathway.^†^Non-peer-reviewed pre-print. #st. = number of studies covered (— where not a count-based synthesis). Values in bold denote the present review.

The primary research question is: what is the current landscape of deployed and research-stage AI-based developmental-screening tools for children aged 72 months or younger, with respect to modality, diagnostic performance, regulatory status, demographic equity, and implementation readiness?

## 2 Materials and methods

### 2.1 Protocol, eligibility, and sources

This scoping review follows Joanna Briggs Institute methodology and the PRISMA-ScR checklist (Tricco et al., 2018); the protocol was registered on the Open Science Framework prior to charting. Inclusion criteria were (i) publication between 1 January 2020 and 15 June 2026; (ii) participants aged 72 months or younger at study entry; (iii) an explicit AI/ML/deep-learning screening or risk-prediction component; (iv) English-language peer-reviewed primary studies; and (v) reporting of at least one diagnostic-accuracy metric (sensitivity, specificity, AUC, PPV, NPV) or a prospective real-world deployment outcome. Reviews, regulatory documents, and foundational epidemiological/guideline references were retained separately for context and comparison but were not charted as primary tool studies. Exclusion criteria covered conference abstracts without a full paper, editorials/letters, method/benchmark papers on pre-labeled datasets without a target-population validation, adult or school-aged-only cohorts, and non-peer-reviewed pre-prints. Seven databases were searched (PubMed/MEDLINE, Web of Science Core Collection, Scopus, IEEE Xplore, ERIC, Cochrane CENTRAL, ClinicalTrials.gov), supplemented by citation searching of landmark papers and a hand search of the FDA 510(k)/De Novo database (product code QPF). Full search strings appear in the Supplementary material.

The restriction to English-language reports was pragmatic rather than principled: the review team could not reliably screen, chart, and appraise full texts in languages outside its competence, and no translation resources were available for this unfunded synthesis; in addition, validation studies of AI screening tools are pre-dominantly indexed in English in the seven databases searched. We did not apply this criterion because we regard non-English evidence as less valuable, and we consider the resulting language bias, together with its likely direction, explicitly in the Limitations.

### 2.2 Selection, charting, and synthesis

Records were de-duplicated and screened independently by both authors; full texts were retrieved for all “include”/“maybe” decisions. A charting form captured citation metadata, tool name, modality, target condition, country, sample size and age range, reference standard, diagnostic-accuracy metrics, regulatory status, demographic subgroup reporting, external-validation status, and identified deployment barriers. A descriptive synthesis was produced, organized by modality, performance, equity, and deployment readiness; no formal meta-analysis or critical appraisal was conducted, consistent with PRISMA-ScR expectations.

## 3 Results

### 3.1 Study selection and landscape

**Figure 1:**
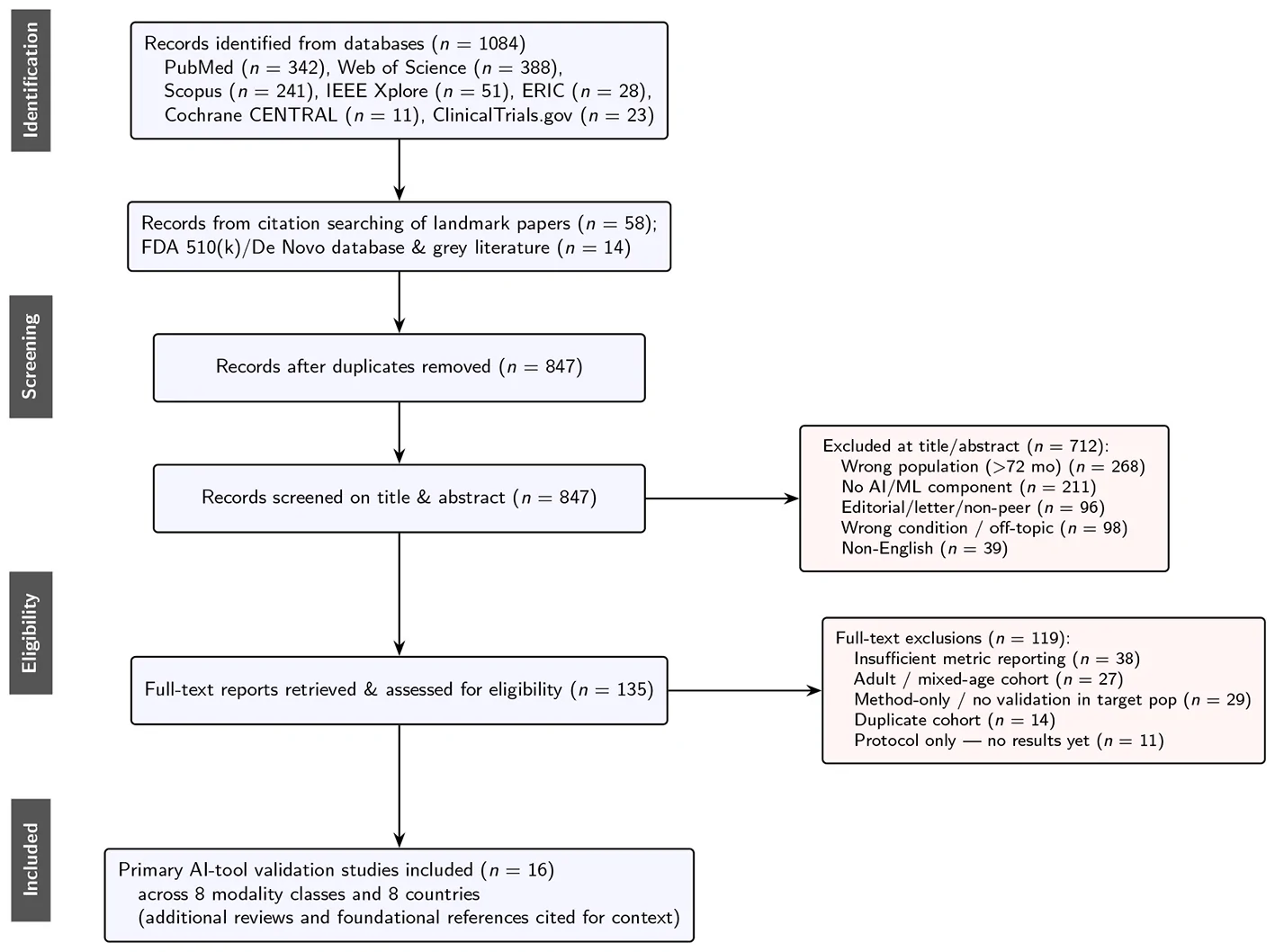
PRISMA-ScR flow diagram for the expanded search (2020–2026).

The PRISMA-ScR flow (Figure 1) summarizes selection. Of 1,084 database records plus 72 from citation/gray sources, 847 remained after de-duplication; 712 were excluded on title/abstract and 135 assessed in full text, yielding 16 primary AI-tool validation studies across eight modality classes and eight countries (Table 2). All charted primary studies targeted ASD or ASD risk; no primary study addressed language-only, ADHD-only, or global-developmental-delay-only screening at comparable maturity.

**Table 2:** Primary AI-based developmental-screening tools included in the scoping review, grouped by modality (16 studies, eight countries).

Tool	Modality	Country	Age (mo)	N	Sens (%)	Spec (%)	Status / AUC
EarliPoint (Jones et al., 2023)	Eye-tracking, social-scene video	USA	16–30	disc./repl.	81.9/80.6	89.9/82.3	FDA 510(k) 2023
Gazefinder (Chetcuti et al., 2024)	Eye-tracking, 2-min protocol	Australia	9–14	54	feas.^a^	—	Commercial (JP)
Gaze metrics (Wang et al., 2024)	Eye-tracking, AVC/FAS/ASC	USA	18–84	39	88–100	80–88	Research (prelim.)
Canvas Dx (Megerian et al., 2022)	Multimodal: caregiver Q + home video + clinician	USA	18–72	—	98.4	78.9	FDA De Novo 2021
Video–audio ens. (Natraj et al., 2024)	Multimodal video + audio NN	Switzerland	12–60	160	86.1^b^	87.5	Research
Joint-Attention DL (Ko et al., 2023)	Computer-vision, joint-attention video	South Korea	24–72	95	99.2	95.5	AUC 0.996
Home-video ID (Kim et al., 2025)	Computer-vision, home video	South Korea	18–48	510	80.0	—	AUC 0.83
Stereotypy det. (Barami et al., 2024)	Computer-vision, movement video	Israel	< 72	241	92.5	—	Research
SenseToKnow (Perochon et al., 2023; Aikat et al., 2025)	Tablet digital phenotyping	USA	17–36^c^	475	87.8	80.8	AUC 0.90
ASDetect (Barbaro and Yaari, 2020)	Smartphone, parent-admin.	Australia	12–24	—	~83	NR	Research
Voice-acoustic (Briend et al., 2023)	Voice / acoustic ML	France	Children	108	91^d^	—	Research
Well-baby EHR (Ben-Sasson et al., 2024)	EHR / ML risk prediction	Israel	0–24^e^	780,610	75.0	81.0	AUC 0.86
Abbrev. M-CHAT-R (Pan et al., 2025)	Questionnaire-based ML	China	Toddlers	6,049	—	—	Research
CHAT-23 LightGBM (Lu et al., 2024)	Questionnaire-based ML	China	18–24	371	90.9	92.2	Research
Vietnamese M-CHAT-R (Van Vo et al., 2026)	Questionnaire-based ML	Vietnam	toddlers	—	—	—	Research
Movement sensors (Wilson et al., 2024)	Wearable sensors	USA	Infants^f^	—	—	—	Research

Metrics are as reported in the source studies and are not directly comparable across heterogeneous designs.

^a^Feasibility/concurrent predictive validity, no fixed sens-spec.

^b^Recall.

^c^Extended to ages 3–8 in replication (Aikat et al., 2025).

^d^Vs. typically-developing children.

^e^Predictors birth–24 month; mean diagnosis ~37.5 mo.

^f^High-likelihood infant cohort. NR, not reported.

### 3.2 Modalities, performance, and geographic breadth

**Figure 2:**
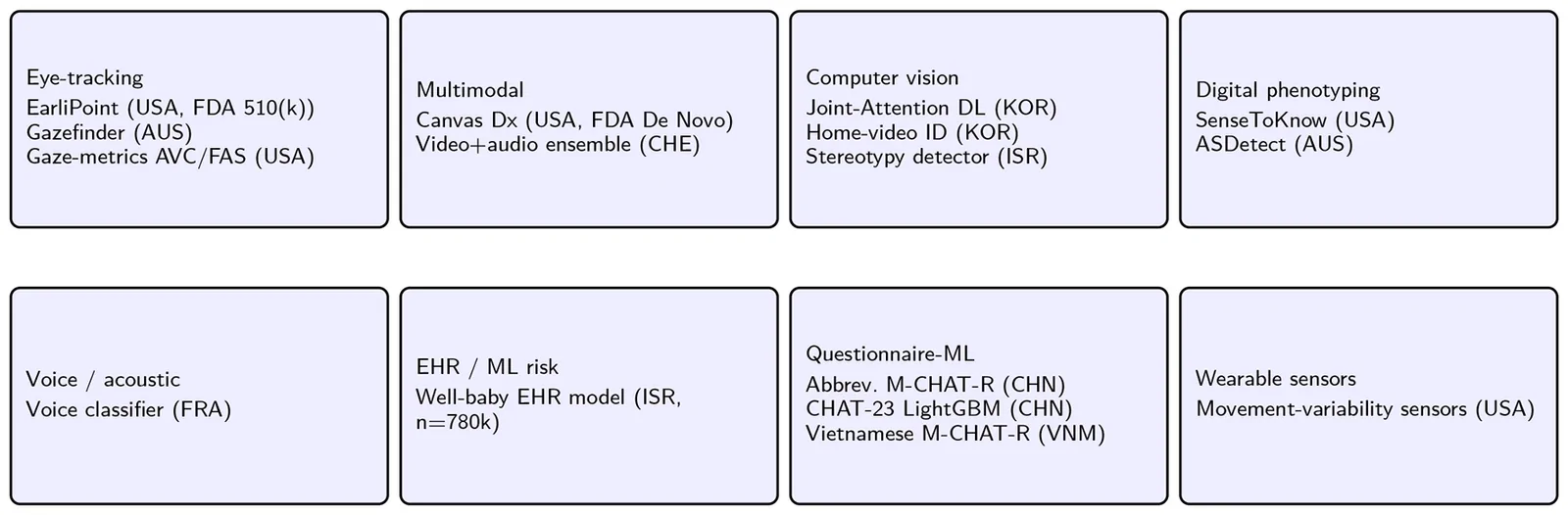
Taxonomy of the 16 included AI-based developmental-screening tools across eight modality classes and eight countries.

The 16 tools span eight modalities (Figure 2 and Table 2). Eye-tracking is represented by the FDA-cleared EarliPoint (Jones et al., 2023), the 2-min Gazefinder protocol (Chetcuti et al., 2024), and novel gaze-metric systems (Wang et al., 2024). Multimodal designs include the FDA-authorized Canvas Dx (caregiver questionnaire plus home video plus clinician inputs) (Megerian et al., 2022) and a video–audio neural-network ensemble (Natraj et al., 2024). Computer-vision behavioral analysis appears as joint-attention deep learning (Ko et al., 2023), automated home-video identification (Kim et al., 2025), and stereotyped-movement detection (Barami et al., 2024). Tablet/smartphone digital phenotyping is represented by SenseToKnow (Perochon et al., 2023; Aikat et al., 2025) and the parent-administered ASDetect app (Barbaro and Yaari, 2020). Newer modality classes, absent from our earlier mapping, include voice/acoustic classification (Briend et al., 2023), large-scale EHR risk prediction (Ben-Sasson et al., 2024), questionnaire-based ML on abbreviated instruments (Pan et al., 2025; Lu et al., 2024; Van Vo et al., 2026), and wearable movement sensing (Wilson et al., 2024).

**Figure 3:**
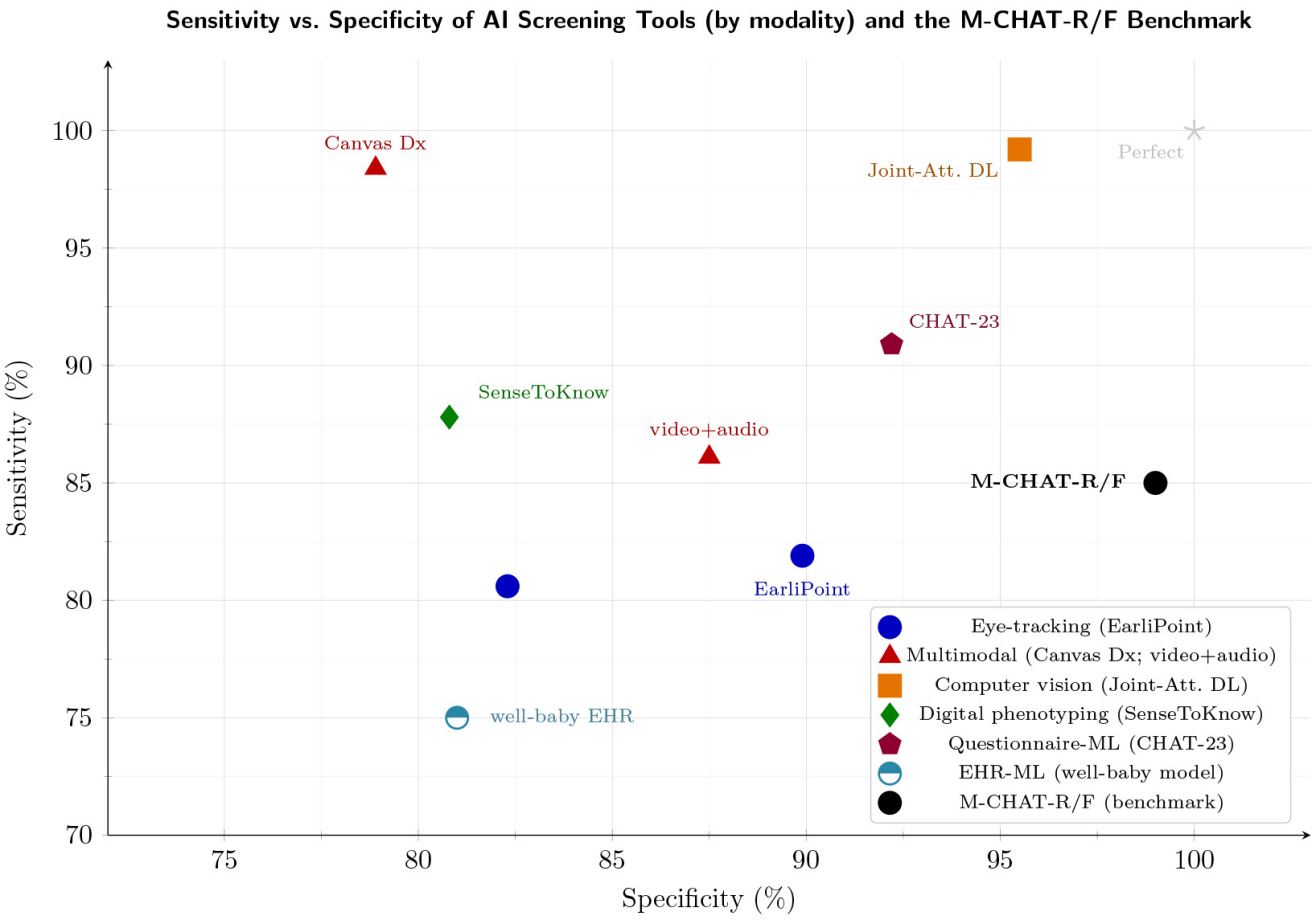
Sensitivity vs. specificity for the AI tools reporting both metrics, colored by modality, against the M-CHAT-R/F benchmark.

Reported sensitivities ranged from approximately 75% [well-baby EHR model (Ben-Sasson et al., 2024)] to 99.2% (joint-attention DL Ko et al., 2023), and specificities from 78.9% [Canvas Dx (Megerian et al., 2022)] to 95.5% (joint-attention DL). The highest AUC was 0.996 (Ko et al., 2023), although that system was validated in a single center with 95 children, limiting generalizability. PPV was consistently the weak metric under realistic prevalence, mirroring M-CHAT-R/F (Robins et al., 2014); Figure 3 positions the tools that report both metrics against this benchmark.

**Figure 4:**
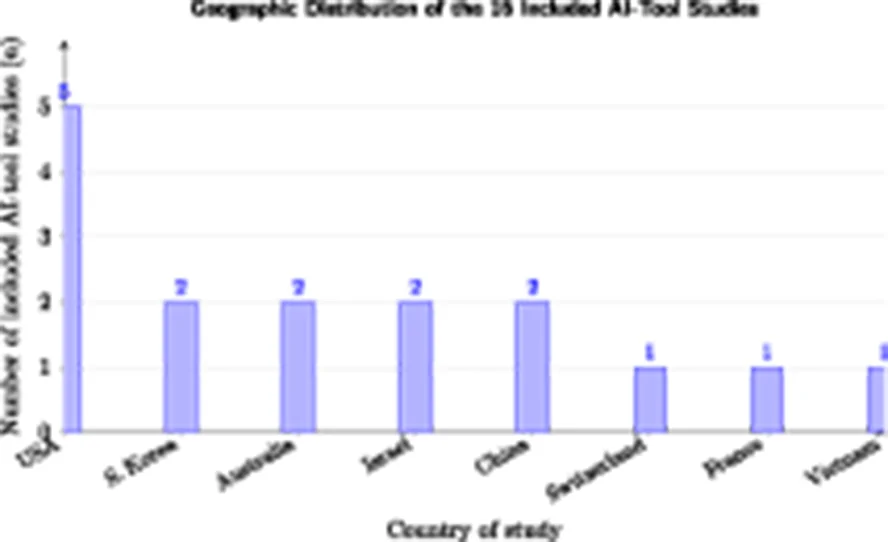
Geographic distribution of the 16 included AI-tool studies by country of study.

These ranges must not be read as a head-to-head comparison of tool performance. The included studies differ fundamentally in design (clinically-referred case–control samples vs. prospective community screening), reference standard (full multidisciplinary diagnostic assessment vs. registry or chart-derived diagnosis), age band, sampling frame, and underlying prevalence—all of which materially shape sensitivity, specificity, and especially PPV. A case–control design that contrasts already-diagnosed autistic children with typically-developing controls will systematically report higher apparent accuracy than the same tool applied to an unselected community population, an effect known as spectrum bias. Consequently, the differences visible in Table 2 and Figure 3 reflect study design at least as much as intrinsic tool quality, and the figures should be read as landscape descriptors rather than as a ranking. This pattern is itself informative: the highest accuracies came from small, single-site, case–control designs, whereas the largest (Ben-Sasson et al., 2024) (*n* = 780, 610) and the most prospectively deployed (Perochon et al., 2023) studies reported more modest, more realistic figures. Geographically, the evidence base now extends well beyond the United States, adding South Korea, Australia, Switzerland, France, Israel, China, and Vietnam, though sub-Saharan Africa, South Asia, the Middle East, and Türkiye remain unrepresented by any primary tool study (Figure 4).

### 3.3 Equity and subgroup validation

Demographic subgroup reporting was the single weakest dimension across the corpus (Table 3). Only SenseToKnow explicitly reported stable accuracy across sex, race, and ethnicity (Perochon et al., 2023). The well-baby EHR model reported sex-stratified performance (boys-only AUC 0.82) (Ben-Sasson et al., 2024), and EarliPoint's replication cohort was more demographically diverse than its discovery cohort, though per-group AUCs were not reported (Jones et al., 2023). The remaining tools provided either unstratified demographics or none. This matters because diagnostic disparities by race, ethnicity, sex, and socioeconomic status are well-documented (Pham et al., 2022; Aylward et al., 2021), female autism is systematically under-detected (Cruz et al., 2024), and the clinical-AI fairness literature converges on inadequate subgroup-stratified validation as the central unmet need (Liu et al., 2025; Huang et al., 2022). An AI screener trained and validated without stratification risks encoding and amplifying these disparities.

**Table 3:** Demographic equity and validation reporting across included tools (✓, reported/stratified; P, partial or noted without stratified metrics; —, not reported).

Tool	Sex	Race/ ethnicity	SES/language	External / prospective validation
SenseToKnow (Perochon et al., 2023)	✓	✓	—	✓ (prospective, well-child visits)
Well-baby EHR (Ben-Sasson et al., 2024)	✓	P	—	✓ (population-scale cohort)
EarliPoint (Jones et al., 2023)	—	P	—	✓ (independent replication cohort)
Canvas Dx (Megerian et al., 2022)	—	P	—	P (pivotal trial)
Home-video ID (Kim et al., 2025)	P^a^	—	—	Multi-site (nine hospitals)
Joint-Attention DL (Ko et al., 2023)	—	—	—	— (single center, *n* = 95)
Voice-acoustic (Briend et al., 2023)	—	—	—	— (single sample)
Questionnaire-ML (Pan et al., 2025; Lu et al., 2024; Van Vo et al., 2026)	—	—	—	Internal cross-validation only

Only one of 16 tools reported fully stratified performance.

^a^Male pre-dominance (>2:1) noted; female/older-child generalizability flagged as a limitation. Remaining tools (Gazefinder, gaze metrics, stereotypy detector, ASDetect, wearables) reported no demographic stratification.

### 3.4 Regulatory trajectory and deployment readiness

**Figure 5:**
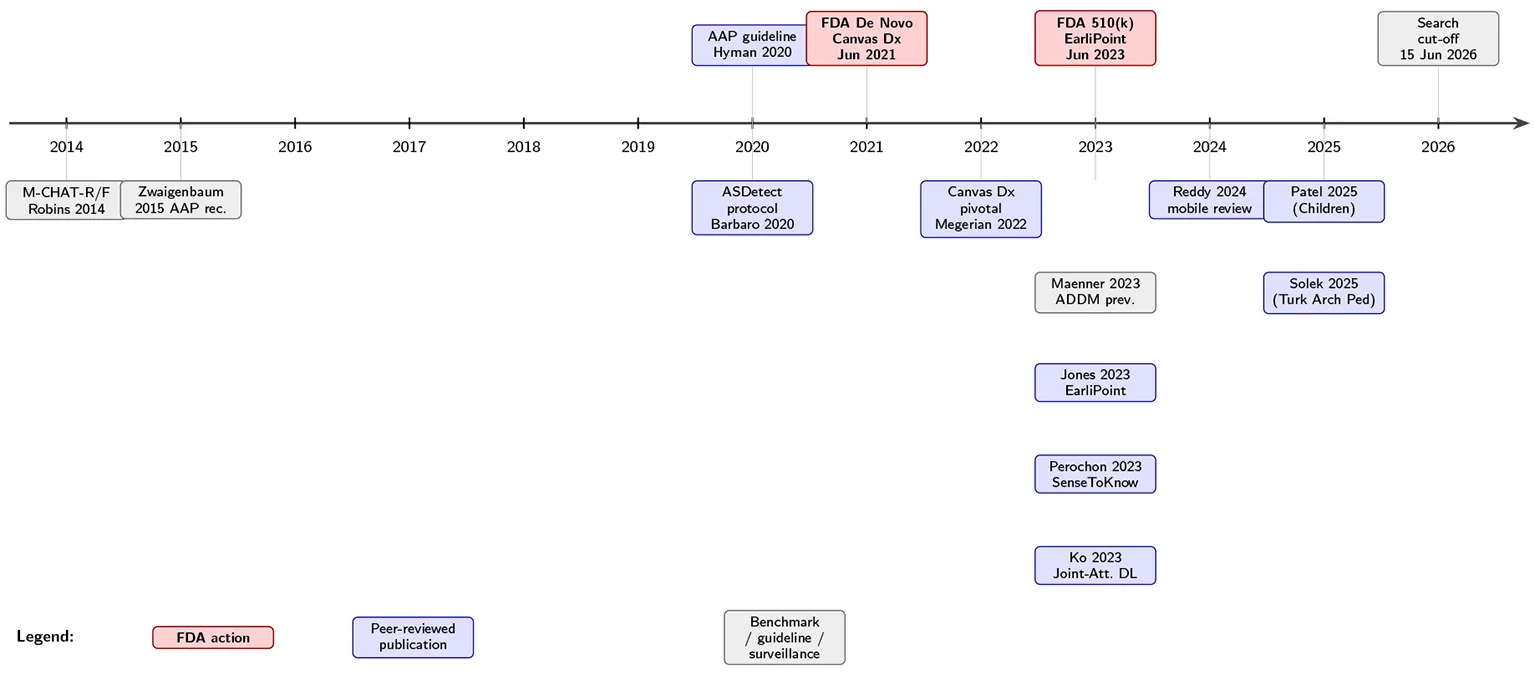
Regulatory and publication timeline of AI-based developmental-screening tools and reference guidelines, 2014–2026.

The regulatory and publication trajectory is summarized in Figure 5. Two FDA authorizations anchor the period: Canvas Dx (De Novo, 2021) and EarliPoint (510(k), 2023), both under software-as-a-medical-device (SaMD) pathways increasingly shaped by the FDA AI/ML action plan and pre-determined change-control guidance (Singh et al., 2025). In the European Union, AI medical devices fall under the Medical Device Regulation and the emerging AI Act (Balogun et al., 2024), yet no CE-marked autism-specific AI screening device was identified in the peer-reviewed literature, a notable transatlantic asymmetry. Beyond regulatory status, deployment readiness varies widely: EarliPoint requires specialty eye-tracking hardware; Canvas Dx yields a determinate output in roughly one third of referred cases; tablet and smartphone tools (SenseToKnow, ASDetect) are the most scalable but report the most modest PPV under community prevalence. Most research-stage tools have not undergone external or prospective validation, echoing broader pediatric-AI findings that few models reach routine workflows (Wang et al., 2025; Katonai et al., 2025).

### 3.5 How this review differs from recent syntheses

Table 1 positions our review against ten recent syntheses. Prior reviews are either modality-bounded, method-aggregating, technology-broad, or generative-AI-specific; none combines deployed-tool case studies with a structured equity matrix, a deployment-readiness lens, and a low-resource guidance-center implementation pathway.

## 4 Discussion

### 4.1 Summary and comparison with the traditional workflow

This review maps a field that has broadened substantially in modality and geography, from five tools across five modalities in early mappings to sixteen tools across eight modalities and eight countries here, yet remains concentrated on ASD and constrained by single-site, case–control validation. AI tools can equal or exceed questionnaire screeners on sensitivity, and sometimes objectivity and scalability, but the binding constraints are generalizability, equity, and operational integration rather than peak accuracy. No tool yet replaces universal M-CHAT-R/F at the 18–24-month visits; the realistic near-term role is a second-line, tiered aid consistent with American Academy of Pediatrics surveillance (Hyman et al., 2020).

### 4.2 Methodological quality and risk of bias in the included evidence

Consistent with PRISMA-ScR and Joanna Briggs Institute guidance, scoping reviews map the extent and nature of evidence and do not require formal critical appraisal, and we therefore did not score the included studies with an instrument such as QUADAS-2. Nonetheless, charting the corpus made several recurring methodological limitations visible, and these should temper any reading of the accuracy figures reported above. First, and most consequentially, most tools were validated in relatively small, single-site, case–control samples that contrast already-diagnosed autistic children with typically-developing controls; such designs are well recognized to overstate accuracy relative to the unselected populations in which screening actually occurs (spectrum bias). Second, external and prospective validation remains rare: only SenseToKnow (Perochon et al., 2023) was evaluated prospectively in routine well-child visits, and only EarliPoint (Jones et al., 2023) reported an independent replication cohort, while most research-stage tools report internal cross-validation only, leaving overfitting and site-specific tuning unquantified. Third, reference standards are heterogeneous, ranging from full multidisciplinary diagnostic assessment to registry- or record-derived diagnosis, which shifts both the target condition and the apparent error rates. Fourth, the two FDA-authorized devices were evaluated in industry-sponsored pivotal trials in which the developer had a financial interest—an established source of optimism bias in diagnostic-accuracy research—and reporting of thresholds and subgroup outcomes was frequently selective. Finally, demographic subgroup reporting was near-absent (Table 3), so the generalizability of the reported metrics to the populations most affected by diagnostic disparities is largely unverified.

Taken together, these features suggest that the corpus, while promising, sits at a relatively early stage of diagnostic-accuracy evidence, and that the highest reported figures are the least likely to survive prospective community deployment. A formal appraisal—for example QUADAS-2 for the diagnostic-accuracy studies and PROBAST for the prediction models—applied within a future systematic review would be a valuable and, we would argue, now necessary next step for this field.

### 4.3 Equity as the priority gap

A central promise of AI screening is to reduce disparities by providing low-cost, objective tools independent of parent literacy or specialty-clinic access. The evidence is only partly aligned with that promise: SenseToKnow is the strongest equity exemplar (Perochon et al., 2023), but most tools report no stratified accuracy. Because diagnostic disparities and female under-detection are well documented (Pham et al., 2022; Aylward et al., 2021; Cruz et al., 2024) and clinical-AI fairness reviews repeatedly flag missing subgroup validation (Liu et al., 2025; Huang et al., 2022), we argue that stratified-accuracy reporting (by sex, race, ethnicity, primary language, and socioeconomic status) should be a default requirement for AI-screening trials, alongside child-centered ethical safeguards (Chng et al., 2025).

### 4.4 Regulatory and data-governance considerations

The FDA SaMD framework now accommodates adaptive AI through pre-determined change-control plans (Singh et al., 2025), while the EU MDR/AI Act imposes high-risk-device obligations (Balogun et al., 2024). The absence of any CE-marked autism screening device suggests regulatory and market pathways outside the United States remain immature. Pediatric deployment further requires strict data governance: in the United States the Children's Online Privacy Protection Act, in the EU the General Data Protection Regulation provisions for minors, and in Türkiye the Personal Data Protection Law (KVKK, No. 6698). Notably, KVKK contains no child-specific provision (parental-consent obligations derive from the Civil Code), a gap relevant to any RAM-based deployment and to broader recommendations for pediatric AI data use (Muralidharan et al., 2023).

### 4.5 Implementation and adoption

Analytic accuracy is necessary but insufficient for adoption. Barriers identified across health-AI implementation reviews include clinician training and AI literacy, workflow and EHR integration, reimbursement uncertainty, trust, and accountability (Hassan et al., 2024; Katonai et al., 2025), and adoption is best understood through implementation-science frameworks such as NASSS (Greenhalgh et al., 2017). A child-development monitoring review likewise found acceptance and outcome evidence to be thin (Reinhart et al., 2024). SenseToKnow's deployment during well-child visits (Perochon et al., 2023) is an instructive exemplar of feasibility, but real-world effectiveness studies remain scarce.

### 4.6 Türkiye/RAM context: a low-resource implementation pathway

Türkiye's child-development pipeline involves the Ministry of National Education's pre-primary curriculum, the Family-and-Social-Services-Ministry Guidance and Research Centers (RAM), and Health-Ministry well-child clinics. AI screening tools are not yet routinely deployed in this pipeline, and only one Turkish-led synthesis on AI for ASD was identified (Solek et al., 2025). RAM centers, publicly funded, nationally distributed, and already tasked with developmental assessment and educational placement, are a natural locus for a pragmatic, equity-oriented pilot: a tablet digital-phenotyping or questionnaire-ML tool, culturally and linguistically adapted to Turkish, with prospective stratified-accuracy reporting. Such a pilot would simultaneously address the three gaps this review identifies (deployment evidence, equity validation, and non-Western/low-resource generalizability) and serve as a transferable template for other middle-income guidance-center systems.

### 4.7 Limitations

Four limitations qualify this review. First, and most importantly for interpretation, the search was restricted to English-language peer-reviewed primary studies. This restriction carries a language bias whose direction is predictable rather than random: tools developed and validated in non-English-speaking settings—for example in China, Japan, Latin America, and Türkiye—are disproportionately likely to be reported in national-language journals and in databases we did not search, and are therefore under-represented in our map. Because one of our central findings is the geographic concentration of the evidence base, this bias may partly manufacture the very pattern we report, and the true modality and geographic breadth of the field is likely wider than Figure 4 suggests. Two considerations partially mitigate, but do not remove, this concern: several included tools originate from non-English-speaking countries (South Korea, Switzerland, France, Israel, China, Vietnam) yet were published in English, and regulatory milestones are documented in English-language registries. Future reviews should nonetheless search national-language sources (e.g., CNKI, SciELO, J-STAGE, ULAKBİM TR Dizin) with native-language strings, and we would expect such a search to soften—though probably not overturn—our conclusion about geographic concentration.

Second, we did not perform formal critical appraisal, consistent with PRISMA-ScR; the methodological concerns we identified are therefore descriptive rather than scored (see Section 4.2). Third, all charted primary studies target ASD, so AI work on language, motor, and ADHD precursors falls outside the mapped scope. Fourth, the reported metrics derive from heterogeneous designs, reference standards, age bands, and base rates, and are not directly comparable across tools; the performance ranges should be read as landscape descriptors, not as pooled estimates or a ranking.

## 5 Conclusion

The 2020–2026 evidence base for AI-based developmental screening of young children has matured in modality breadth and geographic reach: two FDA-authorized devices and a growing set of research prototypes span eye-tracking, multimodal, computer vision, digital phenotyping, voice, EHR, questionnaire-ML, and wearable approaches across eight countries. Yet the field remains autism-concentrated, equity-blind, and weakly translated into routine primary care, with no CE-marked autism screener and near-absent subgroup-stratified validation. Clinicians, child-development practitioners, and policymakers should treat current tools as emerging second-line aids that complement, not replace, validated questionnaire screeners, while the research community prioritizes equity validation, deployment-readiness reporting, and pragmatic implementation studies in low-resource guidance-center settings such as Türkiye's RAM network.

## Supplementary material

Data Sheet 1 Full database search strategies, PRISMA-ScR charting framework, and the initial and final generative-AI prompts.
