# Supplementary material for: Artificial-intelligence–based developmental screening for pre-school children (ages 3–6): a scoping review of deployed tools, clinical validity, equity, and implementation in guidance-center settings
Source: Front Psychol. 2026 Aug 20;17:1913332. doi: 10.3389/fpsyg.2026.1913332 (PMC13539307; doi:10.3389/fpsyg.2026.1913332)
Supplement: Supplementary file 1 [file Data_Sheet_1.pdf]

Supplementary Material: AI-Based Developmental Screening for Preschool Children — Search Strategy and Charting Framework

1 SUPPLEMENTARY METHODS: FULL SEARCH STRATEGIES

The master strategy was developed for PubMed/MEDLINE and adapted to the syntax of each of the remaining databases. Searches covered 1 January 2020 to 15 June 2026 and were restricted to English-language records. The conceptual blocks were: (A) artificial-intelligence/machine-learning methods, (B) developmental screening / autism / neurodevelopmental targets, and (C) early-childhood population.

1.1 PubMed/MEDLINE (master strategy)

```
((("artificial intelligence"[Title/Abstract])
OR ("machine learning"[Title/Abstract])
OR ("deep learning"[Title/Abstract])
OR ("convolutional neural network*"[Title/Abstract])
OR ("computer vision"[Title/Abstract])
OR ("digital phenotyp*"[Title/Abstract])
OR ("eye tracking"[Title/Abstract] OR "gaze tracking"[Title/Abstract])
OR ("voice"[Title/Abstract] OR "acoustic"[Title/Abstract] OR "speech"[Title/A
OR ("wearable*"[Title/Abstract] OR "sensor*"[Title/Abstract])
OR ("electronic health record*"[Title/Abstract] OR "EHR"[Title/Abstract])
OR ("smartphone"[Title/Abstract] OR "mobile application*"[Title/Abstract]
OR "tablet"[Title/Abstract]))
AND
(("developmental screening"[Title/Abstract])
OR ("autism"[Title/Abstract] OR "autistic"[Title/Abstract] OR "ASD"[Title/Abs
OR ("developmental delay"[Title/Abstract])
OR ("language delay"[Title/Abstract])
OR ("ADHD"[Title/Abstract])
OR ("global developmental"[Title/Abstract]))
AND
(("preschool*"[Title/Abstract] OR "toddler*"[Title/Abstract]
OR "early childhood"[Title/Abstract] OR "infant*"[Title/Abstract] OR "child"[
AND ("2020"[Date - Publication] : "2026"[Date - Publication])
AND English[Language]
```

1.2 Web of Science Core Collection

```
TS=((("artificial intelligence" OR "machine learning" OR "deep learning"
OR "convolutional neural network*" OR "computer vision" OR "digital phenotyp*
OR "eye tracking" OR "gaze" OR voice OR acoustic OR speech OR wearable*
OR sensor* OR "electronic health record*" OR smartphone OR tablet) AND ("deve
```

```
screening" OR autism OR autistic OR ASD OR "developmental delay" OR "language
delay" OR ADHD OR "global developmental") AND (preschool* OR toddler* OR
"early childhood" OR infant* OR child))) refined by Publication Years 2020--2
and Language English.
```

1.3 Scopus

```
TITLE-ABS-KEY(({artificial intelligence} OR {machine learning} OR {deep
learning} OR {computer vision} OR {digital phenotyping} OR {eye tracking}
OR voice OR acoustic OR wearable* OR {electronic health record} OR smartphone
OR tablet) AND (autism OR {developmental screening} OR {developmental delay}
OR ADHD) AND (preschool* OR toddler* OR infant* OR child)) AND PUBYEAR >
2019 AND PUBYEAR < 2027 AND LANGUAGE(english).
```

1.4 IEEE Xplore

```
("All Metadata":"machine learning" OR "deep learning" OR "computer vision"
OR "eye tracking" OR "digital phenotyping") AND ("All Metadata":autism OR
"developmental screening" OR "developmental delay") AND ("All Metadata":presc
OR toddler OR infant OR child); filtered 2020--2026.
```

1.5 ERIC

```
(("artificial intelligence" OR "machine learning" OR "computer vision" OR
"eye tracking") AND (autism OR "developmental screening" OR "developmental
delay" OR "early identification") AND (preschool OR toddler OR "early childho
2020--2026.
```

1.6 Cochrane CENTRAL

```
("artificial intelligence" OR "machine learning" OR "deep learning") AND
(autism OR "developmental screening") AND (preschool OR toddler OR child);
2020--2026.
```

1.7 ClinicalTrials.gov

```
Condition/disease: autism OR "developmental delay"; Other terms: "artificial
intelligence" OR "machine learning" OR "digital" OR "eye tracking"; Ages:
Child (birth--17); first posted 2020--2026.
```

2 SUPPLEMENTARY NOTE: CHARTING FRAMEWORK (PRISMA-SCR)

Each included primary study was charted on the following variables: citation metadata; tool/device name; modality class; target condition; country of study; sample size and age range; reference standard; reported diagnostic-accuracy metrics (sensitivity, specificity, AUC, PPV, NPV); regulatory status (FDA De Novo / 510(k); CE-mark); demographic subgroup reporting (sex, race, ethnicity, socioeconomic status, language); external/prospective validation status; and identified deployment barriers. The descriptive synthesis was organized by modality, diagnostic performance, demographic equity, and deployment readiness, consistent with the PRISMA-ScR reporting framework. No formal risk-of-bias appraisal or meta-analysis was performed, in keeping with scoping-review methodology.

3 SUPPLEMENTARY NOTE: GENERATIVE-AI PROMPTS

In accordance with the journal's generative-AI policy, the tool used was **Claude** (Anthropic; model Claude Opus 4.8; `https://www.anthropic.com`), which assisted with drafting and language editing of the text and with generating the LaTeX/TikZ code for the figures. The model was directed iteratively across the project; the initiating and concluding prompts are reproduced below. All output was reviewed and verified by the authors, and every reference was independently checked against its DOI.

3.1 Initial prompt

"Draft a PRISMA-ScR scoping review of artificial-intelligence-based developmental-screening tools for preschool children (ages 3–6), following Joanna Briggs Institute methodology. Chart the AI tools by modality, diagnostic performance, regulatory status, equity, and practitioner adoption. Verify every reference against its DOI and never fabricate any citation."

3.2 Final prompt

"Strengthen and format the review for Frontiers: expand the evidence base to all deployed and research-stage AI screening tools across modalities and countries (all DOIs Crossref-verified); add a structured equity matrix, a deployment-readiness analysis, a comparison with prior reviews, and data figures; reframe the discussion around equity and low-resource (guidance-center) implementation; and produce the manuscript in the official Frontiers template."
